# Sociodemographic aspects and health care-related outcomes: a latent class analysis of informal dementia care dyads

**DOI:** 10.1186/s12913-021-06708-6

**Published:** 2021-07-23

**Authors:** Henrik Wiegelmann, Karin Wolf-Ostermann, Werner Brannath, Farhad Arzideh, Jan Dreyer, Rene Thyrian, Liane Schirra-Weirich, Lisa Verhaert

**Affiliations:** 1 Institute for Public Health and Nursing Research, Health Sciences Bremen, University of Bremen, Grazer Straße 4, 28359 Bremen, Germany; 2 Competence Centre for Clinical Trials, University of Bremen and Clinic Bremen-Mitte, Bremen, Germany; 3 German Centre for Neurodegenerative Diseases (DZNE), Site Witten, Germany; 4 Department of Social Services, Centre for Participation Research, Catholic University of Applied Sciences of North Rhine-Westphalia, Cologne, Germany

**Keywords:** Dementia, Informal caregivers, Dementia care dyads, Latent class analysis, Caregiver burden, Quality of life, Stability of care arrangements, Caregiver health, Health care service use

## Abstract

**Background:**

Studies revealed the importance to assess dementia care dyads, composed of persons with dementia and their primary informal caregivers, in a differentiated way and to tailor support services to particular living and care circumstances. Therefore, this study aims first to identify classes of dementia care dyads that differ according to sociodemographic, care-related and dementia-specific characteristics and second, to compare these classes with regard to healthcare-related outcomes.

**Methods:**

We used data from the cross-sectional German DemNet-D study (*n* = 551) and conducted a latent class analysis to investigate different classes of dementia care dyads. In addition, we compared these classes with regard to the use of health care services, caregiver burden (BIZA-D), general health of the informal caregiver (EQ-VAS) as well as quality of life (QoL-AD) and social participation (SACA) of the person with dementia. Furthermore, we compared the stability of the home-based care arrangements.

**Results:**

Six different classes of dementia care dyads were identified, based on best Bayesian Information Criterion (BIC), significant likelihood ratio test (*p* <  0.001), high entropy (0.87) and substantive interpretability. Classes were labelled as “adult child parent relationship & younger informal caregiver”, “adult child parent relationship & middle aged informal caregiver”, “non family relationship & younger informal caregiver”, “couple & male informal caregiver of older age”, “couple & female informal caregiver of older age”, “couple & younger informal caregiver”. The classes showed significant differences regarding health care service use. Caregiver burden, quality of life of the person with dementia and stability of the care arrangement differed also significantly between the classes.

**Conclusion:**

Based on a latent class analysis this study indicates differences between classes of informal dementia care dyads. The findings may give direction for better tailoring of support services to particular circumstances to improve healthcare-related outcomes of persons with dementia and informal caregivers.

**Supplementary Information:**

The online version contains supplementary material available at 10.1186/s12913-021-06708-6.

## Background

Every year about 10 million dementia diagnoses are confirmed worldwide and estimates suggest that the number of persons with dementia will almost triple until 2050 and then reach about 150 million [1]. In European countries, the need for care and support is increasing simultaneously, largely provided by informal caregivers in home-based care arrangements [2]. This also applies to Germany, where estimates predict the proportion of the population in need of long-term care to increase by 40–70% until 2050. The majority (approx. 70%) of persons with dementia are living at home and are cared for by informal caregivers [3].

The potentially overwhelming care-related demands and their adverse health consequences are well documented. Informal caregivers of people living with dementia face multiple disadvantages in physical, psychological, social and economic domains [4–7]. Furthermore, it has been shown that individual domains might reinforce each other negatively. This relates, for instance, to the transmission effect of stigmatization on the perceived psychological burden, when social disadvantages lead to psychological problems [8]. This may also be the case for the interaction between economic and health problems. Another example is chronic stress triggered by long-term financial problems and its adverse health consequences. Therefore, tailored psychosocial support services are of crucial relevance to ease health problems of informal caregivers, to promote quality of life and social inclusion of people with dementia and to strengthen home-based care arrangements [9–11].

The role of the dyadic relationship structure between persons with dementia and their caregivers received more attention in recent years. Due to the mutual influence both individuals have on each other, it seems to be reasonable and effective to design psychosocial support in a dyadic manner rather than focusing on one of the two individuals separately [12, 13]. Furthermore, research suggests that dyadic structures of home-based care arrangements are heterogeneous and differ with respect to socio-demographic, relational and regional aspects of the dyadic relationship. Those variations in the dyadic structures can cause significant differences in relevant clinical outcomes for both individuals [14–21]. Based on these findings it is reasonable to suggest that there are typical structural features in dyadic relationship constellations and that meaningful subgroups of dyads can be identified along these features. A multi-dimensional representation of dementia dyad subgroups would offer the opportunity to take a differentiated view on life and care situations and to further develop psychosocial support structures in a tailored approach. However, a multidimensional target-group-specific tailoring of support programs to particular subgroups of dyads has hardly been implemented in practice yet [22, 23]. The relatively small effects measured so far for psychosocial interventions may be due to insufficiently targeted interventions to subgroups of persons with dementia and their informal caregivers [14, 22]. Thus, approaches that focus on particular subgroups might be more effective than so-called one size fits all interventions [22, 24, 25]. Overall, there is still a lack of knowledge about the heterogeneity of dyadic relationship constellations in home-based dementia care settings. With the German DemNet-D study, data on a large number of community-dwelling dyads of informal caregivers and persons with dementia in Germany was collected in the years 2012 to 2015. Using a model-based clustering procedure, the present study aims to provide an evidence-based analysis of subgroups of dementia care dyads.

An increasingly popular method in quantitative health and health care research for identifying subgroups in data sets is latent class analysis (LCA). LCA is a cross-sectional latent variable mixture modeling approach and serves as an effective procedure to identify a small set of underlying subgroups characterized by multiple dimensions, which may differ for instance in their needs for support [26–28]). In LCA the term subgroup is often used as synonym for the term class. The approach has already shown its potential in caregiving research, for example to investigate different classes of service users [29, 30], caregiving experiences classes [31] or caregiver profiles [32].

The aims of the present study are a) to identify different classes of dementia care dyads in order to develop and describe a multidimensional typology and b) to investigate differences between the classes with regard to relevant healthcare-related outcomes. The study intends to contribute to the ongoing discourse on the adequate tailoring of psychosocial interventions to certain subgroups of persons living with dementia and informal caregivers [22, 33, 34].

## Methods

### Sample and data

The analysis was performed using cross-sectional data from the DemNet-D study (baseline survey). This multidimensional and multidisciplinary evaluation study (2012–2015) investigated the care and living situation of community-dwelling persons living with dementia and their informal caregivers as service users of 13 regional dementia networks (DCN) in Germany. Ethical approval was obtained from the ethics committee at Ernst-Moritz Arndt University in Greifswald (registration number BB 107/12). The present study includes data from 551 community-dwelling dyads (551 persons with dementia, 551 informal caregivers). Originally, the DemNet-D data set contains 560 dyads. We excluded nine of these dyads because professional legal guardians and other formal caregivers provided information. Table 1 summarizes the inclusion criteria for the study participants.

**Table 1:** Inclusion criteria for participants of the DemNet-D study

Person with dementia	Informal caregiver
− Living at home − Dementia diagnosis (formally diagnosed by a medical professional or reported by the caregiver) − Informal caregiver present − Utilization of 1 of the 13 regional dementia care networks included in the study	− Primary responsibility as informal caregiver (family member, friend, or neighbor) of a person with dementia − Receives no payment for his/her support other than constant attendance allowance − able to provide detailed information about the person with dementia

Both, persons with dementia and the informal caregiver had to submit written informed (proxy) consent to participate in this study. Participants were identified and contacted by staff of the dementia care networks. More detailed descriptions of the original study population and the data collected have already been provided in previous publications [35, 36].

### Measures

*Persons living with dementia:* We used the Functional Assessment Staging (FAST), designed to assess the stage of dementia (Score: 1–7). The higher the value, the more severe the dementia [37]. To assess the intensity of agitated behavior in people with dementia we used the CMAI-D (Cohen Mansfield Agitation Inventory). With the CMAI-D 30 typical behaviors of persons with dementia are surveyed, recording their frequency (Score: 1 never - 7 several times per hour) [38]. Everyday competencies were assessed using the IADL (Instrumental Activities of Daily Living) scale (Score 0–8). A higher value indicates a higher degree of autonomy [39]. Furthermore, we used socio-demographic indicators, including age, gender and social class. The latter, using information by the person with dementia, was operationalized according to the Scheuch-Winkler Index [40, 41].

*Informal caregiver:* As sociodemographic indicators we used age, gender and occupation (full-time, part-time, none). The relational indicators are the informal care relationship (couple, adult-child, other/non-kinship) and the living situation (living together, not living together but close, other/further away). Furthermore, we included the size of the informal support network (number of other persons involved in care and support). As care-related indicators we used the time (mean hours) informal caregivers spent for care and support on an average day, an item taken from RUD (Resource Utilization in Dementia) questionnaire [42] as well as the duration of care (years/months).

*Region:* External data was linked via postal codes to examine potential influences of non-personal, regional factors. To analyze regional socioeconomic inequalities in health we integrated the German Index of Socioeconomic Deprivation (GISD), which uses regional data (education, occupation, income) to group regions into the three deprivation groups high, middle and low [43]. Furthermore, we created two quotas as proxies for regional nursing and medical infrastructures. For the nursing quotient, we used the number of persons in need of care per employee in outpatient care services per district or city and the national mean value to group districts or cities with a below average or above average rate. The medical quotient was calculated based on the number of contract physicians and psychotherapists per 100.000 inhabitants. Again, we applied the national mean value to form a group of regions above the average and another group below the average. Data was acquired from the German Federal Statistical Office, the Federal Institute for Research on Building, Urban Affairs and Spatial Development as well as the Federal Register of Physicians.

### Outcome measures

#### Persons living with dementia

To assess the quality of life of persons living with dementia, the QoL-AD (Quality of Life in Alzheimer’s Disease) was applied, using the proxy measures provided by informal caregivers (Score: 13–52). A higher value indicates a better QoL [44]. Furthermore, the Sense of Acceptance in Community Activities (SACA) tool was used to assess the social inclusion of persons living with dementia. SACA is based on self-assessment by the person with dementia (Score: 8–32). Higher values indicating better social inclusion [45].

#### Informal caregivers

Subjective caregiver burden was measured with four subscales of the field version of the Berlin Inventory of Caregivers’ Burden with Dementia Patients (BIZA-D PV) designed for counselling services [46]. The four subscales ‘burden due to cognitive losses’, ‘burden due to aggression/resistance’, ‘personal constraints/health’ and ‘lack of social recognition’ were used instead of a global scale to be able to detect different pathways of perceived caregiver burden. In addition, for the present study the visual analogue scale (EQ-5D VAS) was used to assess the overall health status of informal caregivers (Score: 0–100). Higher values indicating better health status [47].

#### Care Arrangement

The stability of the home-based care arrangements was assessed by the informal caregivers using a four-level scale (Score: 0–3) developed by von Kutzleben et al. [48]. The caregivers are asked to evaluate their monthly supply situation in general. The scale ranges between the poles “situation is well arranged, no further assistance necessary” and “care at home doesn’t work anymore, transfer to a nursing home is intended”.

#### Service use

We analyzed health care services used (use/no use) to identify potential support opportunities. Therefore, the use of medical (general practitioner, neurologist) and therapeutical (physiotherapist, occupational therapist) services within the last 6 months and the current use of nursing (day-care, outpatient care service, short-term care) and support (support group, visiting service, household help) services was assessed. Furthermore, we examined which sources of information (informal: TV/radio, newspaper, internet, family; medical: general practitioner, medical specialist, pharmacy; nursing: outpatient care service, care counselling centers, care insurance, health insurance, welfare association; civil society: Dementia service centers, Alzheimer’s society, self-help group, citizens’ center/senior citizens’ office, church/religious community) dyads have used so far to gather information about dementia services. If at least one of the services of the respective category was used, the category was rated “use”. The use of sources of information as well as the current use of nursing and support services were measured with items taken from the D-IVA (Instrument for Assessing Home-Based Care Arrangements for People with Dementia) instrument [49]. Single items from caregiver questionnaires of the DemNet-D study were applied to assess the use of medical and therapeutical services within the previous 6 months [41, 50, 51].

### Statistical analysis

To identify subgroups of dementia dyads a latent class analysis (LCA) was performed [52]. LCA is a model-based statistical procedure using item response patterns for classifying individuals into different classes that are as homogeneous as possible. The classification is based on observed variables (or indicator variables). Relationships between values of the indicator variables are attributed to the existence of an unobserved latent variable (categorical latent variable). Classes were evaluated based on criteria indicating a good classification and model fit. First, a low Bayesian Information Criterion (BIC), where lower values indicate better fit. Second, a good relative entropy (>.80), where higher values indicate better classification certainty. Third, a reliable class assignment based on consensus build through discussion within the research team [31]. We used BIC as the main indicator to evaluate model fit and as the main criterion for model selection (selection of number of classes). Additionally, we used the likelihood ratio test to control model selection through BIC. The entropy of the model was also calculated as a model fitting measure. The main criterion for comparing different class solutions, for instance 4-class model vs. 3-class model, was a decreasing BIC value. With the likelihood ratio test, we subsequently controlled, whether the hypotheses that the 3-class model fits the data better as 4-class model can be rejected (*p*-value < 0.05).

Besides the identification of subgroups, latent class analyses allow the examination of the association of a latent variable with other external variables. Given the variables listed in Table 2 and study aims, each external variable was hypothesized to be dependent on the latent classes. To estimate these associations, the three-step procedure was used [53]. We also investigated the dyad classes’ potential association with other dyadic variables given in Table 1. The three-step algorithm consists of the following steps: In step 1 LCA model is run for indicator variables and the model parameters are estimated. In step 2 dyads are assigned to the estimated classes based on their posteriori class membership probabilities and in step 3 potential associations between these classes and external variables are investigated using multinomial logistic regression analysis. The maximum-likelihood estimation was performed with the aforementioned adjusted three-step LCA, which is a modification of the three-step procedure and corrects the bias by using the stepwise estimation [53–55].

**Table 2:** Hierarchy of indicator variables for LCA analysis

Level	Indicator variable
1	
	Age in years informal caregiver
	Age in years persons living with dementia
	Informal care relationship
	Sex informal caregiver
	Sex person living with dementia
	Living situation of dyad
2	
	Size of further informal support
	Occupation informal caregiver
	Social class (Scheuch-Winkler Index)
	Time caregiver spent for care and support (RUD)
	Duration of care (in years)
3	
	Instrumental activities of daily living (IADL)
	Agitated behavior (CMAI)
	Dementia severity (FAST)
4	
	Regional socio-economic deprivation (GISD)
	Regional care and medical infrastructure

### Procedure of latent class analysis

In a first step, we estimated the number of classes, class size and class structure. A hierarchical list (Table 2) of indicator variables formed the basis for this. The hierarchy was developed based on previous research findings [56] as well as study objectives. Variables were added gradually to the model calculations. The first indicator variables (level 1) were used to determine the optimized number of classes and thereby the main characteristics of the classes. This number of classes (*n* = 6) was set despite adding further indicator variables of levels 2–4. Following this procedure, the optimized 6-class model based on primary indicators remained, and further indicators only influenced the class characteristics (probabilities), if they were significant (see Table 5). To reduce the probability of local optima we used the integrated option in Latent Gold 5.1 software and repeated the algorithm with different starting points chosen at random.

The inclusion of lower ranking variables was made after all higher-ranking variables had been tested. Therefore, a first LCA was conducted using the six variables of level 1: sex and age of informal caregiver, sex and age of person with dementia, informal care relationship and the living situation of the dyad. Model solutions were assessed using the BIC criterion, the entropy score as well as a meaningful and coordinated interpretation by the research team. To test whether the influence of individual indicator variables on the model is significant, two statistical tests were used. First, the Wald-Test assesses, if the regression coefficients within all classes are equal to zero (null hypothesis), which would mean that there is no significant difference between the classes with respect to the relevant indicator variable. Second, we applied the Likelihood Ratio Test (LRT). The LRT tests whether the model with six indicator variables represents a significant improvement compared to models with less variables (null hypothesis test). The variables of level 2–4 were subsequently added to the model. If a variable significantly improved the fit of the overall latent class model, it was included; if not, it was not considered (see Table 5). Finally, we examined the BIC and entropy to test, if the final model with six classes and all variables sufficiently fitted to the data. The Expectation-Maximization (EM) algorithm was applied for the maximum likelihood (ML) estimation of the model. The EM algorithm uses no imputation algorithm for missing values. The only assumption is that missing data is missing at random (MAR). We used all observed attributes for each individual case and made no imputation and no removing procedures for missing values. The latent class analysis was performed with the software Latent Gold® version 5.1.

## Results

### Baseline characteristics

We included 551 home-based care dyads in this study. Table 3 shows baseline sociodemographic and clinical characteristics. Overall, the mean age of the persons with dementia is 79.5 years (SD: 8.5 years). More than half of the persons with dementia are female (57%). Median QoL-AD score is 28.7 (SD: 5.5) and the median score for social inclusion, measured with the SACA scale is 24.4 (SD: 4.2). The informal caregivers mean age is 64 years (SD: 12.9), 75% are female. Slightly more than 60 % have no occupation (62.4%). The mean EQ VAS score is 66.4 (SD: 19.6). The perceived burden due to a) cognitive decline is 8.1 (SD: 4.7), b) aggression and confusion is 6.0 (SD: 4.9), c) personal restrictions is 8.3 (SD: 5.8) and d) lack of social support 7.7 (SD: 5.5). Sixty-one percent of the dyads live together, 50.6% have a couple relationship.

**Table 3:** Baseline characteristics of persons living with dementia, informal caregiver, dyads and regional characteristics

**Person with dementia (*n*** **= 551)**		
Mean age	in years	79.5 (8.5)
Sex	female	57% (316)
Social class	low	44.4% (245)
	middle	21.8% (120)
	high	7.1% (39)
	no information	26.7% (147)
Functional abilities (FAST)	1–4	6.6% (36)
	5–7	90.2% (497)
	no information	8.9% (49)
Instrumental activities of daily living (IADL)	0–2	62.7% (345)
	3–8	28.4% (157)
	no information	8.9% (49)
Cohen Mansfield Agitation Inventory (CMAI)		
inappropriate	yes	61.7% (340)
aggressive	yes	15.2% (84)
agitated	yes	58.8% (324)
QoL-AD	(3–52)	28.7 (5.5)
SACA	(1–32)	24.4 (4.2)
**Informal caregiver (*n*** **= 551)**		
Mean age	in years	64 (12.9)
Sex	female	75% (412)
Occupation	full-time	14.3% (79)
	part-time	20.7% (114)
	none	62.4% (344)
	no information	2.5 (14)
Time spent for care	(0-24 h/day)	4.7 (5.4)
Time spent for support	(0-24 h/day)	4.6 (5.1)
Care duration	in years	4.2 (3.6)
EQ VAS	(0–100)	66.4 (19.6)
BIZA-D Subscales; Burden due to …		
cognitive losses	0–16	8.1 (4.7)
aggression/resistance	0–20	6.0 (4.9)
personal constraints/health	0–20	8.3 (5.8)
lack of social recognition	0–24	7.7 (5.5)
**Dyad (*n*** **= 551)**		
Informal care relationship	couple	50.6% (279)
	adult-child	42.1% (232)
	other/non-kinship	6.7%(37)
	no information	0.5% (3)
Living situation	living together	61.2% (337)
	not living together but close	22.3% (123)
	other/further away	1.5% (8)
Further informal support	yes	88% (485)
	no	11.6% (64)
	no information	0.4% (2)
Health care service use: Information sources		
Informal sources	no use	28.9% (159)
Medical sources	no use	35% (193)
Nursing sources	no use	44.8% (247)
Civil society sources	no use	70% (369)
Health care service use: Professional care		
Medical services	no use	8.2% (45)
Therapeutic services	no use	69.7% (384)
Nursing services	no use	41.4% (228)
Support services	no use	68.2% (376)
Stability of care arrangements	0 = care at home does not work anymore	3.3% (18)
	1 = urgent need for more help	7.2% (40)
	2 = if dementia progresses, more help is needed	51.9% (286)
	3 = situation is well arranged	30.3% (169)
	no information	7.3% (34)
**Region**		
Regional socio-economic deprivation	low	7.2% (40)
	middle	68.6% (378)
	high	24.1% (133)
Regional outpatient care infrastructure	–	7.8 (1.9)^a^
Regional medical infrastructure	–	63.5 (6.1)^b^

Categorical data are presented as (%)/N. Continuous data are presented as Mean/(SD)

^a^ Quota: Number of persons in need of care per employee in outpatient care services per district/city

^b^ Quota: Number of contract physicians and psychotherapists per 100.000 inhabitants

### Latent class analysis

Model fit and interpretation suggested that a model with six classes represented the most precise solution (see Table 4). This analysis showed that the 6-class model scores lower (thus better) on the BIC-scale as the models with 1–5 classes. Furthermore, the model with seven classes does not fit the data better than the 6-class model. Overall, the 6-class model showed the best BIC value in combination with a significant likelihood ratio test compared with the 3-class model and a high entropy score (0.87).

**Table 4:** Fit statistics for different latent class models

Class	1	2	3	4	5	6	7
Log-Lik (LL)	− 3460	− 2946	− 2773	− 2743	− 2711	− 2682	− 2672
BIC (LL)	7015	6031	5730	5713	5693	5679	5704
Entropy Score^a^	1.00	0.99	0.99	0.88	0.87	0.87	0.85
LRT (bootstrap-*p*-value)^b^	–	0.00	0.00	0.00	0.00	0.00	0.04

^a^Entropy Score: This value indicates how distinctly the dyads can be assigned to the classes. The closer the value is to 1, the better the model fit

^b^ LRT: Likelihood ratio test for the model l with C classes vs. model with C-1 classes

The latent class analysis yielded a final best-fitting model solution with six classes based on 15 significant indicator variables (*p* <  0.05). Table 5 shows all indicator variables included in the final model, their respective *p*-value (Wald-Test) and the corresponding determination coefficient R^2^. For further characterization of the dyad classes, we used only the indicator variables whose variance was best explained by the model (*R*^*2*^ ≥ 0.3, in bold in Table 5): Sex and age of the person with dementia; sex, age and occupation of the informal caregiver; the informal care relationship and the dyadic living situation.

**Table 5:** Significant indicator variables of the final 6-class model (p- and R^2^ values)

Indicator	***p***-value (Wald-Test)	R^**2**^
Sex person living with dementia	< 0.001	**0.689**
Sex informal caregiver	0.012	**0.534**
Age person with dementia	< 0.001	**0.333**
Age informal caregiver	< 0.001	**0.721**
Informal care relationship	< 0.001	**0.874**
Living situation of dyad	< 0.001	**0.586**
Further informal support	< 0.001	0.109
Occupation of informal caregiver	< 0.001	**0.514**
Social class	< 0.001	0.144
Time informal caregiver spend for care and support	< 0.001	0.111
Instrumental activities of daily living (IADL)	< 0.001	0.105
Agitated behavior (CMAI)	0.001	0.045
Dementia severity (FAST)	0.044	0.037
Regional care quota	0.026	0.104
Regional medical quota	0.003	0.138

Table S1 Additional file 1 summarizes the best fitting 6-class model solution based on most likely class membership.

A crucial step in interpreting the results of latent class analyses is to label and describe the statistically identified different classes concisely, using the decisive indicator variables of the model. In this study, the process involved successive discussions within the project team. The next section focuses on describing the key class-building indicators of the final 6-class model solution, based on most likely class membership. Class differences are also shown in Table S1 (Additional file 1).

### Class-building indicators

Class 1 (class size: 22.9%) is labeled as “adult-child-parent relationship & younger informal caregiver”. These dyads are characterized by intergenerational child-parent care relationships. The caregivers of this subtype are relatively young with a mean age of 50.8 years. The persons with dementia have a mean age of 79.5 years. The dominant gender ratio is female, with women as informal caregivers of their mothers or grandmothers living with dementia. Typically, care arrangements are characterized by spatial distance between caregivers and the persons with dementia (17.1% live together). A large proportion of informal caregivers (80.3%) have a job and work part-time or full-time.

Class 2 (class size: 17.1%) is labeled as “adult-child-parent relationship & middle-aged informal caregiver”. Like the dyads of class 1, class 2 dyads are characterized by intergenerational child-parent relationships typically consisting of a female daughter/daughter-in-law providing care and support for her mother/mother-in-law living with dementia. The professional activity of the caregivers is less pronounced compared to class 1. With a mean age of 60.1 years, the caregivers of these dyads are almost 10 years older than caregivers of class 1 and can be described as a group “around retirement age”. The mean age of the persons living with dementia is 87.2 years, which is by far the highest mean age among all classes. In difference to class 1 and class 3, nine out of 10 dyads (86%), live together or close to each other. The close spatial relationship of both individuals is a defining feature of class 2 dyads.

Although class 3 is small in terms of size (8.8%) this class stands out due to its specific dyadic constellation. We labeled class 3 “non-family relationship & younger informal caregiver” to emphasize the fact that a majority of these dyads have a non-family informal care relationship (53.7%). Even if this is only a small majority, this class clearly stands out from the other classes. The age structure in this class indicates intergenerational relationship constellations (difference: approx. 30 years). The caregivers are on average slightly over 50 years (Mean: 51.9) and thus comparatively young. The persons with dementia have a high mean age above 80 years (Mean: 81.8). The gender ratio of this class is predominantly female; in about 8 out of 10 cases, both individuals are female. The level of employment is pronounced (83.2% part-time or full-time). Most of the dyads do not live together in the same household (6.1% living together).

Class 4 (Class size: 14%) is labeled as “couple & male informal caregiver of older age”. The dyads live in intragenerational spouse/couple relationships. Informal caregivers of these dyads are male with a mean age of 78.4 years. The persons with dementia are female and with a mean age of 77.2 years slightly younger than their male partners. The higher age of both individuals is associated with the lower employment level of caregivers (4% in part-time jobs). The typical living situation of this class is the shared household (99.9%).

Class 5 (Class size: 31.4%), labeled as “couple & female informal caregiver of older age”, typically comprises intragenerational care relationships with female caregivers of advanced age caring for their male (spouse) partner living with dementia. The caregivers have a mean age of 73.8 years and the persons with dementia are slightly older with a mean age of 78.7 years. Along with advanced age, only few caregivers of this class are employed (0.7% part-time). The shared household is the standard for these dyads (98.8%).

**Fig. 1:**
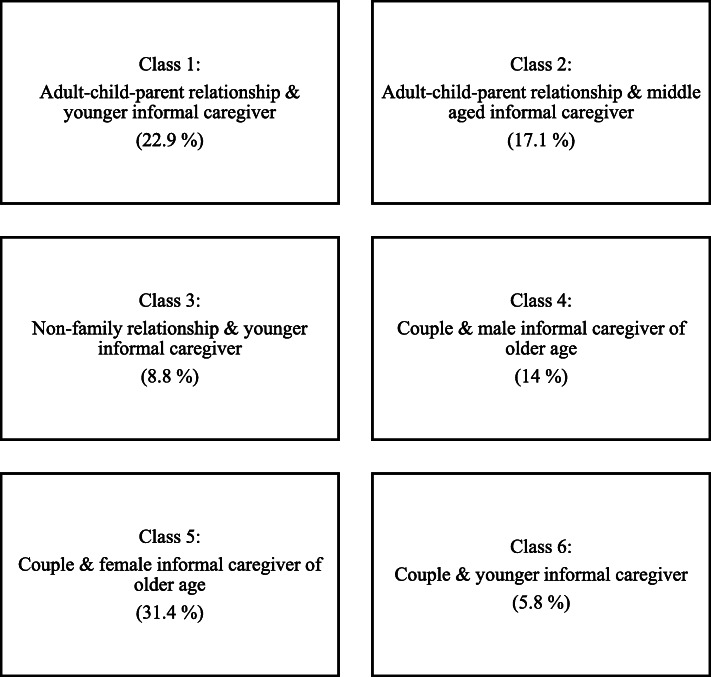
Six dyad classes identified via LCA (Percentage of total sample in parentheses)

Class 6 (5.8%), labeled as “couple & younger informal caregiver”, is a class characterized by intragenerational couple relationships at a younger age. The gender ratio is mixed, with a majority of female caregivers and male persons with dementia. The mean age of the informal caregivers is 57.6 years. With a mean age of 64.1 years, persons with dementia of this class are relatively young on average, indicating a group of persons with young onset dementia. The typical living situation of these dyads is the shared household (96.5%). Many of the caregivers of this group are employed either part-time (43.6%) or full-time (31.5%). Figure 1 provides an overview of the six classes identified (*n* = 551).

### Associations with outcome measures

Following the identification of the best fitting LCA class solution, the associations between the six classes and several outcome measures were investigated.

#### Use of information sources

There are no significant differences between the dyad classes in terms of using nursing care services as an information source for care-related issues. Similarly, no significant differences in the use of civil society sources can be observed. The use of medical information sources as well as the use of informal information sources differs significantly between the six classes a (*p*-value = 0.01, Wald-Test) (see Table 6).

**Table 6:** Association between classes and use of information sources and health care services

Outcome measures	Classes						***p***-value (Wald-Test)
	1	2	3	4	5	6	
*Class size (in %)*	*22.9*	*17.1*	*8.8*	*14.0*	*31.4*	*5.8*	
Information source use: Informal (%)	86.5	65.8	74.2	62.4	68.4	57.6	0.01
Information source use: Medical (%)	66.9	51.3	55.1	68.1	73.6	58.8	0.016
Information source use: Nursing care (%)	57.5	66.8	57.8	44.8	51.0	55.3	0.10 (n.s.)
Information source use: Civil society (%)	29.8	32.3	28.5	21.8	40.4	41.8	0.068 (n.s.)
Health care service use: Medical (%)	95.2	89.4	75.2	96.2	93.2	93.1	0.005
Health care service use: Therapeutical (%)	23.7	20.5	35.3	28.7	36.3	49.1	0.018
Health care service use: Nursing care (%)	58.3	80.6	90.0	43.6	47.5	44.2	< 0.001
Health care service use: Support services (%)	31.7	26.4	48.6	37.6	30.6	14.5	0.067 (n.s.)

*n.s.* not significant

#### Health care service use

There are no significant differences between the classes with respect to the use of support services. Significant differences between the classes can be observed for medical services, therapeutical services and nursing care services. In general, it can be shown that the use of nursing care services is more prevalent in adult child-parent relationships (classes 1, 3) and non-family relationship (class 3) than in couples (classes 4, 5, 6) (see Table 6).

#### Quality of life (Qol-AD) of persons with dementia

The QoL of persons living with dementia, rated by the informal caregivers, differs significantly between the classes (*p*-value< 0.001, Wald-Test). Persons living with dementia of classes 4, 5 and 6, who live in intragenerational couple relationships with their informal caregiver, show a significantly higher quality of life than persons living with dementia of classes 1, 2 and 3. The lowest value occurs for caregivers in class 2, which simultaneously express relatively high caregiver burden.

#### Social inclusion of persons with dementia (SACA)

Concerning the perceived social inclusion of persons living with dementia (self-assessment) no significant differences between the six different classes could be observed.

#### Health status of informal caregivers (EQ. 5D VAS)

The general health status of the informal caregivers is worse in the intragenerational spousal relationships of classes 4, 5 and 6 than in classes 1 and 2 with adult-child relationship or class 3 with a non-family relationship. Furthermore, it is significantly worse (*p*-value < 0.001, Wald-Test) than in classes 1 and 3 with the youngest groups of caregivers.

#### Caregiver Burden (BIZA-D subscales)

Subjective caregiver burden differs significantly between the six classes (*p*-values < 0.001, Wald-Test). The informal caregivers in class 6 and 2 feel most burdened. In contrast, the caregivers in class 3 indicate rather low levels of subjective burden. The burden scores for the other classes are mainly in the moderate range.

#### Stability of care arrangement

The subjectively perceived stability of the home-based care arrangements differs significantly between the six classes (*p*-value = 0.016, Wald-Test). In class 3, informal caregivers rated the situation as more stable compared to other classes, especially class 1, 2 and 6. Table 7 below summarizes results regarding the clinical outcome measures.

**Table 7:** Comparison of clinical outcome measures based on most likely class outcome

Outcome measures	Classes						***p***-value (Wald-Test)
	1	2	3	4	5	6	
Class size (in %)	22.9	17.1	8.8	14.0	31.4	5.8	
Quality of life persons living with dementia (QoL-AD, 3–52)^a^	28.8	26.2	27.2	30.1	29.6	29.7	< 0.001
Social inclusion of persons living with dementia (SACA, 1–32)	24.1	24.2	25.4	23.2	24.9	24.7	0.23
Subjective caregiver burden due to … (BIZA-D PV)							
cognitive losses (0–16)	7.7^b^ (0.46)	9.6 (0.55)	3.7 (0.74)	7.5 (0.36)	8.7 (0.35)	9.4 (0.81)	< 0.001
aggression and resistance (0–20)	5.3 (0.44)	7.3 (0.70)	2.7 (0.59)	5.5 (0.63)	6.8 (0.42	7.2 (1.04)	< 0.001
personal constraints/health (0–20)	6.7 (0.58)	10.5 (0.70)	6.6 (1.14)	8.9 (0.42)	7.2 (0.63)	10.7 (1.07)	< 0.001
lack of social recognition (0–24)	7.1 (0.48)	9.4 (0.61)	8.6 (1.06)	6.0 (0.68)	7.0 (0.42)	11.1 (1.02)	< 0.001
Health status informal caregiver (EQ VAS, 0–100)	75.2	65.1	74.9	61.7	61.0	62.4	< 0.001
Stability of care arrangement (0–3)	2.0	2.1	2.4	2.2	2.3	2.0	0.016

^a^ The ranges of the scales used are listed in brackets following the names of the corresponding instruments. The maximum scores are underlined. Interpretation: Qol-AD: The higher the value, the better the quality of life; SACA: The higher the value, the better the social inclusion; BIZA-D subscales: The higher the values, the higher the burden; EQ VAS: The higher the value, the better the health status; Stability of care arrangement: The higher the value, the more stable the care arrangement

^b^ Class values presented here are mean values. In parentheses: Standard deviation (SD)

## Discussion

In this study, LCA was applied to a large, German sample of persons living with dementia and their informal caregivers. The analyses aimed to a) identify different classes of dementia care dyads in order to develop and describe a typology and to b) investigate differences between the classes with regard to relevant healthcare-related outcomes. Six meaningful classes of dementia care dyads were identified and differences between them have been examined regarding healthcare-related outcome measures.

### Identification of dementia care dyad classes

Informal caregivers and persons living with dementia are often treated as a relatively homogeneous group in intervention research, while their diversity regarding personal, relational and other aspects characterizing life and care situations remain largely invisible [22, 25, 57, 58]. This study shows that informal dementia care dyads differ in terms of factors related to individual persons (gender, age, occupation) as well as to relational factors (informal care relationship, living situation). Our results suggest that these differences among care dyads should be carefully reviewed in the design and implementation of counselling and support services.

The key characteristics distinguishing the classes from each other are care relationship between informal caregiver and persons living with dementia (spousal relationship, child-parent relationship, non-family relationship) and age of the informal caregivers (younger age, middle age, older age). In addition, the dyad classes differ significantly with regard to gender relations, the living situation and the occupation status of the informal caregivers. Several of these indicators have also been identified as relevant distinguishing characteristics in previous research. For instance, with regard to the age of the informal caregivers [18], the dyadic gender relation [59], the kinship relationship [14], the living situation [20] or the occupational status of informal caregivers [15]. However, the present study goes beyond the findings of the above-mentioned studies by exploring the dyadic constellation in a multifactorial way and analyses the characteristics not isolated but in a combined multivariate and model-based latent class analysis.

### Comparing outcome measures between classes

Statistically significant differences between the identified dyad classes can be observed both in terms of health care service use as well as in terms of other important outcomes. Regarding the dimensions of health care service use, the use of medical information sources and the use of informal information sources differed significantly between the six classes (see Table 6). Further significant differences were observed in the quality of life of persons living with dementia. Assessed by the informal caregivers, persons living with dementia of classes living in spousal relationships (4, 5, 6), show a higher quality of life than persons living with dementia in adult child-parent dyads (classes 1, 2, 3) do. The lower QoL-AD assessment in classes 1, 2 and 3 might be due to the higher mean age of the persons with dementia, which is in turn common for adult-child informal care relationships [14]. For a progressive condition such as dementia, even if there is no direct causality, typically more severe symptoms arise with very old age (80 years and older), associated with increasing limitations in everyday life [60]. These factors in turn have an influence on health-related assessments made by proxies like informal caregivers [61]. It is noticeable that the informal caregivers in classes 4, 5 and 6 rate their own health status relatively poorly, but at the same time the QoL of persons with dementia were rated highest in these classes. Other studies point to the inconsistent evidence on the influence of proxy health status and assessment of QoL in people with dementia [62]. The results of this study do not provide a clear if and how this might be linked directly.

The health status (EQ VAS) of the informal caregivers is lower in the intragenerational spousal relationships (classes 4, 5, 6) than in the intergenerational adult child-parent relationships (classes 1, 2, 3), and significantly lower than in classes 1 and 3 with the youngest informal caregivers. This is consistent with the results of Pinquart et al. [14], who examine differences between spouses, children and children-in-law in their meta-analysis and find that spouses report greater physical burden than children and children-in-law in the same role. Norton et al. [63] explain those differences inter alia by the own medical co-morbidity, greater risk for functional limitations, and greater likelihood of fatigue at physical exertion. In this context, however, the higher age of the informal caregiver should not be ignored, which often goes hand in hand with the spousal relationship. Studies show that informal caregiver in spousal relationships are usually much older than caring children/children-in-law of persons living with dementia [14]. A large age difference between these informal care relationships is also evident in this study. Most dyads assessed in classes 4, 5 and 6 live together in one household, which in turn could make it more difficult for informal caregivers to distance themselves from the care situation and take care of their own health.

The subjective burden of informal caregiver also differs significantly between the six classes. The informal caregivers of class 6 (couple & younger informal caregiver) and class 2 (adult-child-parent relationship & middle-aged informal caregiver) perceive the most severe burden but on different pathways. Based on BIZA-D PV subscales used, class 6 caregivers express relatively strong burden due to personal constraints and a lack of social recognition. This subgroup is made up of younger couples that have to deal with dementia at a very early stage of life. Caregivers in those informal care relationships report high levels of stress and burden due to the necessity to readjust work and care (reducing working hours or quitting their job) and redefine the intimate spousal relationship [64, 65]. The dementia diagnosis occurs early in life when it is not expected, which can lead to a financial and psychosocial crisis for the family system [64, 65]. For class 2, other mechanisms for increased burden might occur. The persons living with dementia in this group are by far the oldest in comparison with other subgroups. Here, severe dementia-related symptoms are often responsible for higher levels of burden, since informal caregivers have to provide extensive efforts to ensure care [66]. The caregivers of class 3 with non-family relationships and younger informal caregiver, on the other hand, indicate comparatively low levels of burden. One explanation for this could be indirect relief effects of so-called “legitimate excuses” [67]. Legitimate excuses reflect social norms that are widely accepted by society and internalized by acting persons. In the context of informal care, for instance, geographical distance and professional activity are among the legitimate excuses for being less confronted with expectations to take over care tasks. This again may account for the comparatively low burden values of the class 3 and the relatively high values of class 5 and 6, both couple-dyads living in the same household. Similarly, male gender represents a socially accepted legitimate excuse, because men are usually less likely to be confronted with society’s expectation of having to provide care for the elderly [67]. The differences in burden between classes 4 and 5 can also be seen against this background. In three out of four subscales, the reported burden values for female informal caregivers of class 5 are higher than in class 4 with male caregivers. This is despite the fact that both subgroups are very similar in other respects. However, another reason for the differences between men and women could be that men are less likely to report burden, which might be due to gendered coping strategies where men are more likely to negate stress or negative feelings [68].

There are also significant differences between the six different dyad classes for the assessment of the stability of the care arrangements. For instance, the stability of the care arrangements was rated better in in class 3 with non-family relationship and younger informal caregivers compared to other classes, especially class 1 (adult-child-parent relationship & younger informal caregiver) and class 6 (couple & younger informal caregiver). A reason for the lower stability scores in class 6 might be the increased psychosocial stress, reflected in the stated subjective caregiver burden. This relationship has already been described [69]. Despite positive ratings for both caregiver burden and their own general health status, the caregivers in class 1 perceive their care arrangement as relatively unstable. This seems somehow contradictory but cannot be explained by the variables investigated in this study. A current meta-study aims to explain the complex phenomenon of stability of home-based care arrangements for persons living with dementia and could help to better understand this contradictory finding [70].

### Limitations

Some factors limited the findings of this study. Although a large data set was used, it was not based on a random sample and therefore has some limitations with regard to the statistical generalizability. However, a comparison with other surveys in Germany shows that the DemNet-D study represents a valid sample of home-based dementia patients in Germany in terms of basic sociodemographic characteristics, but differs in terms of, for instance, subjective burden of the caregivers or use of health care services [35]. Since we conducted a secondary analysis, we had to deal with possible restrictions due to the original data, (i.e., self-assessment measures, sophistication of variables). Further research on informal dementia care dyads should also add relational indicators, such as relationship quality, relationship closeness or positive and negative dyadic interactions, as they are relevant for coping behaviors that in turn moderate health related outcomes [7, 71]. As we used only cross-sectional data, future research should add a longitudinal perspective; i.e. to analyze changes in clinical outcomes within different dyad subgroups [10]. This would be important to take into account the progressive quality of care processes [48]. It should also be noted that the data used for the analysis was collected in 2012 and thus is about 10 years old.

## Conclusion

It is crucial for researchers in the field of dementia care to be able to identify subgroups of dyadic constellations within the overall population, which have similar patterns of life and care situations. This approach allows to further investigate specific interrelations, e.g. which subgroups of dementia dyads are commonly found in the general population, how prevalent they are, what characterizes them, what future bio-psycho-social health outcomes can be predicted, and whether those outcomes change over time. A latent class perspective can provide important insights about key underlying population subgroups, to subsequently tailor prevention and treatment as well as psychosocial support programs to their needs.

The typology developed here allows a multidimensional description and thus a more comprehensive representation of dyadic subgroup constellations in home-based dementia care. The results indicate the need to approach dyad subgroups specifically in terms of counseling, service use promotion and easing of caregiver burden. Recent research findings on counselling services in the care and nursing context highlight the need for a comprehensive assessment of care situations and to derive support services needed [72].

Health and social care providers can also benefit from the results of this study. For instance, they can provide a basis for the development of a screening tool to assess specific living and care situations based on typical dyadic care constellations right after a diagnosis. With only a few indicators to be collected, it would be possible to carry out an early initial classification to identify specific dyadic constellations in a time-saving manner. This selection step could be implemented in existing support and counseling structures with little effort and could be carried out flexibly. The results might then be used to describe type-specific risk profiles regarding the health and social situation of dementia care dyads. Derived from this, preventive and relieving support services could be offered.

There is no uniform group of dementia care dyads. Dyads rather differ significantly with regard to certain individual, relational and social aspects that lead to differences in relevant outcomes. These differences must be identified and target group-specific interventions to improve the quality of life and reduce the perceived burden of informal caregivers must be made available. A one-size-fits-all approach runs the risk of being too unspecific.

## Supplementary Information

**Additional file 1.** This file contains Table. It summarizes the best fitting 6-class model solution based on most likely class membership.

## Data Availability

The data that support the findings of this study are available from the corresponding author, HW, upon reasonable request.

## Abbreviations

DemNet-D: Evaluationsstudie von Demenznetzwerken in Deutschland
BIZA-D: Berlin Inventory of Caregivers’ Burden with Dementia Patients
EQ-VAS: EuroQoL Group Visual Analogue Scale
QoL-AD: Quality of Life in Alzheimer’s Disease
QoL: Quality of Life
SACA: Sense of Acceptance in Community Activities
BIC: Bayesian Information Criterion
LCA: Latent Class Analysis
DCN: Regional dementia networks
FAST: Functional Assessment Staging
CMAI-D: Cohen Mansfield Agitation Inventory
IADL: Instrumental Activities of Daily Living
RUD: Resource Utilisation in Dementia
GISD: German Index of Socioeconomic Deprivation
D-IVA: Instrument for Assessing Home-Based Care Arrangements for People with Dementia
LRT: Likelihood Ratio Test
EM: Expectation-Maximization
ML: Maximum likelihood
MAR: Missing at random
SD: Standard deviation
n.s.: not significant
