# Supplementary material for: Sociodemographic aspects and health care-related outcomes: a latent class analysis of informal dementia care dyads
Source: BMC Health Serv Res. 2021 Jul 23;21:727. doi: 10.1186/s12913-021-06708-6 (PMC8299572; doi:10.1186/s12913-021-06708-6)
Supplement: Supplementary file 1 — Additional file 1. This file contains Table. It summarizes the best fitting 6-class model solution based on most likely class membership. [file 12913_2021_6708_MOESM1_ESM.docx]

|  | **Classes** | | | | | |
| --- | --- | --- | --- | --- | --- | --- |
|  | **1** | **2** | **3** | **4** | **5** | **6** |
| **Label** | **Adult-child - parent**  **relationship & younger informal caregiver** | **Adult-child-parent relationship & middle aged informal caregiver** | **Non-family relationship & younger informal caregiver** | **Couple & male informal caregiver of older age** | **Couple & female informal caregiver of older age** | **Couple & younger informal caregiver** |
| **Class size (%)** | 22.9 | 17.1 | 8.8 | 14.0 | 31.4 | 5.8 |
| **Informal caregiver, female (%)** | 78.6 | 86.8 | 79.5 | 0.9 | 100 | 63.9 |
| **Persons living with dementia, female (%)** | 87.0 | 87.1 | 78.0 | 99.9 | 0.3 | 42.6 |
| **Mean age informal caregiver** | 50.8 | 60.1 | 51.9 | 78.4 | 73.8 | 57.6 |
| **Mean age person living with dementia** | 79.5 | 87.2 | 81.8 | 77.2 | 78.7 | 64.1 |
| **Informal care relationship (%):** |  |  |  |  |  |  |
| Couple | 0.7 | 0.6 | 0.0 | 99.9 | 99.9 | 93.1 |
| Adult-Child | 94.7 | 93.7 | 46.3 | 0.1 | 0.1 | 6.9 |
| Other/Non-kinship | 4.6 | 5.7 | 53.7 | 0.0 | 0.0 | 0.0 |
| **Living situation (%):** |  |  |  |  |  |  |
| Living together | 17.1 | 40.0 | 6.1 | 99.9 | 98.8 | 96.5 |
| Living nearby | 47.9 | 46.1 | 36.5 | 0.1 | 1.2 | 3.5 |
| Other/further away | 35.0 | 13.9 | 57.4 | 0.0 | 0.0 | 0.0 |
| **Occupation informal caregiver (%):** |  |  |  |  |  |  |
| No | 19.7 | 67.1 | 16.8 | 95.9 | 99.3 | 24.9 |
| Part time | 42.5 | 28.1 | 41.3 | 4.1 | 0.7 | 43.6 |
| Full time | 37.8 | 4.8 | 41.9 | 0.0 | 0.0 | 31.5 |

Table S1 Characteristics of 6-class model based on most likely class membership (n=551)
